# Development of a fluorescence–based multiplex genotyping method for simultaneous determination of human papillomavirus infections and viral loads

**DOI:** 10.1186/s12885-015-1874-9

**Published:** 2015-11-06

**Authors:** Zhengrong Sun, Rong Zhang, Zhonghua Liu, Chao Liu, Xiulin Li, Weiqiang Zhou, Lianxia Yang, Qiang Ruan, Xu Zhang

**Affiliations:** 1 Virus Laboratory, The Affiliated Shengjing Hospital, China Medical University, Shenyang, Liaoning 110004 China; 2 Jiangsu Bioperfectus Technologies Limited Company, Jiangsu, 225300 China

**Keywords:** HPV, BMRT, GenoArray test, Sequence, Cervical lesion

## Abstract

**Background:**

Persistent high-risk human papillomavirus (HPV) infection is correlated with an increased risk of developing intraepithelial lesion or malignancy (NILM). The aims of the current study is to establish a method named BioPerfectus Multiplex Real Time (BMRT) HPV assay for simultaneous typing and quantifying HPVs, and to evaluate it by comparison with HPV GenoArray test and PCR-sequencing method, as well as histological status.

**Methods:**

A total of 817 cervical specimens were evaluated by BMRT method and HPV GenoArray test, using PCR-sequencing method as the reference standard; simultaneously, high-risk HPV-16 and -18 DNA loads were assessed in 443 specimens to investigate the correlation with infection outcomes.

**Results:**

The overall detection coincidence rate between BMRT assay and HPV GenoArray test is 96.6 % and the Kappa value is 0.760. In addition, the sensitivity and positive predictive value of BMRT is 98.4 % and 95.7 % compared with the results detected by PCR-sequencing method, respectively. HPV-16 viral load has a correlation with CINs or worse lesions. By comparing with infected women presenting NILM /cervicitis, the cutoff value for HPV-16 from patients with CINs was 0.827. With this cutoff value, 74.6 % sensitivity and 72.5 % specificity for prediction of HPV-16 infected patients with CINI and higher CIN were achieved. High significance was obtained when comparing the infected women presenting NILM/cervicitis with women either with CIN and cervical carcinomas (*p* < 0.001).

**Conclusions:**

The BMRT assay seemed to be a good alternative approach for HR-HPV testing, due to its high level of automation and ability to quantify HPV-16, HPV-18 and other HR-HPVs.

## Background

Development of cervical cancer is usually related to an infection with human papillomavirus (HPV), especially with any of the 12 high-risk genotypes (HR, HPV-16, −18, −31, −33, −35, −39, −45, −51, −52, −56, −58 and −59) [1–4]. HPV-16 and HPV-18 are the most common genotypes found in more than 70 % of cervical cancer patients, in which HPV-16 can be detected in more than 50 % cases [5, 6]. It seems that HPV-16 is not only more common, but also more oncogenic [7]. Co-infection with multiple HPV types is common [8, 9]. Studies show a tendency of some genotypes to cluster, and some genotypes to be inversely associated [10–12]. The biological significance of the individual infection in a multiple infection is however, difficult to establish. But, there is an association between multiple infections and increased risk of neoplasia compared to single infections [8, 13, 14].

HPV viral load, as a product of the number of infected cells and the number of virus per infected cell, is therefore influenced by two main factors: the extent of an HPV infection on the cervical surface and the level of viral production in the infection area. Viral load has been suggested to be a potential biomarker for cervical intraepithelial neoplasia grade II (CINII) or higher CIN. However, there is no consistent evidence that a one-time measurement of viral load is a useful marker of prevalent disease or disease progression so far [15]. The impact of viral load change has been assessed in only a few studies [16, 17]. An investigation in a hospitalized population of HPV-16 positive and cytologically normal women demonstrated that an increased HPV-16 viral load measured at six month interval was associated with a progress of CINII/III+ in infected women, while decreased viral load over time was more likely to be found in women who remained cytologically normal [17]. Changes in viral load and the associations of the changes with disease risk may imply the complex interaction between HPV and human host, and potentially serve as an additional predictive marker for the outcomes of infection.

At present, the most commercially used method for HPV genotyping is the HPV GenoArray test (HPV GenoArray test kit; Hybribio Ltd, Hong Kong) in China. The method is based on reverse line blot technology (RLB), in which the PCR products are hybridized to HPV type-specific probes on a membrane. In our previously research, HPV GenoArray test have been performed concerning the prevalence and distribution of HPV genotypes in women with cervical lesions from Liaoning Province, China, but the main drawbacks of the assays are its high material cost and its time-consuming performance [18]. In addition, it is difficult to give a diagnosis for borderline cases due to the read-outs being based on direct visualization only. Moreover, quantifications of viral DNA in samples are unavailable by this technique. In the study, the BioPerfectus Multiplex Real Time (BMRT) HPV assay was developed to detect 18 HR-HPV types and 3 low risk (LR) HPV types as well as the viral loads simultaneously, and the clinical value of the BMRT assay was estimated in cervical specimens. The purpose of the present study was to validate the BMRT HPV assay developed for detection of 21 HPVs, including 18 HR-HPV types of HPV-16, −18, −26, −31, −33, −35, −39, −45, −51, −52, −53, −56, −58, −59, −66, −68, −73, −82 and 3 LR-HPV types of HPV-6, −11, −81, by comparing with HPV GenoArray test and to evaluate whether the measurements of HPV-16 and −18 viral loads had potential diagnostic utility by comparing with histological diagnosis.

## Methods

### Clinical samples

Informed consent was obtained from participation in the study and this study was approved by the ethics committee of Affiliated Shengjing Hospital of China Medical University. Specimens were obtained from patients in the Department of Obstetrics and Gynecology of the hospital, who subjected for a routine diagnosis for HPV infection, between July 2011 and November 2012. Clinical data of the patients were collected. For each patient, cervical cells were scraped from the ecto- and endocervix with a cytobrush. The cervical specimens were placed in the PreservCyt® LBC medium (Cytyc, Bedford, MA, USA) and transported to the laboratory, where they were kept at temperatures between 2 °C and 8 °C until performance with a routine HPV GenoArray test. A total of 817 HPV positive cervical samples detected previously, including 467 single positive samples and 350 multiple positive samples, were selected for the present study. Among them, 364 samples were HPV - 16 positive, 142 samples were HPV - 18 positive and 311 samples were positive for other HPV types in the routine laboratory detections. All patients in this retrospective study had liquid based cytology test or colposcopy done at the time the cervical scrapes were taken. The median age of the studied populations was 39 years old (range 18–66 years old) at the time the cervical scrapes were collected.

### DNA preparations

Total cellular DNA from the residual samples was extracted using QIAamp DNA mini kit (Qiagen, Hilden, Germany), according to the manufacturer’s instructions. The concentration of DNA was determined in a spectrophotometer (DU 640, Beckman Coulter). Successful extraction of human genomic DNA was evaluated by amplifying a 258-base pair (bp) fragment of glyceraldehyde 3-phosphate dehydrogenase (GAPDH) gene using primers 5′-AGAAGGCTGGGGCTCATTTG-3′ (forward) and 5′-AGGGG CCATCCACAGTCTTC-3′ (reverse). The PCR reactions were carried out in a thermo- cycler under the following conditions: an initial 95 °C for 9 min; 40 cycles of 94 °C for 20 s, 55 °C for 30 s, 72 °C for 30 s; and a final extension at 72 °C for 5 min. In each PCR assay, negative and positive controls were included. Only DNA preparations, from which the GAPDH DNAs were successfully amplified, were used for further analyses.

### HPV GenoArray test

HPV GenoArray test was performed using HybriMax Kit (Hybribio Limited Corp., China) according to the manufacturer’s instructions. Briefly, HPV specific fragments in the DNA preparations were amplified by PCR, and genotyping for HPVs was done by flow-through hybridization to a gene chip as described previously [18]. The gene chip contains type specific oligonucleotides immobilized on a nylon membrane, including 13 HR-HPVs of HPV-16, −18, −31, −33, −35, −39, −45, −51, −52, −56, −58, −59 and −68, 5 LR-HPVs of HPV-6, −11, −42, −43 and −44, and HPV-53, −66 and -CP8304, which are popular in the Chinese population. The final results were determined by direct visualization of colorimetric changes on the chip.

### BMRT *HPV PCR assay*

In the BMRT HPV PCR assay, PCR primers and corresponding TaqMan probes were designed to detect each of the 21 most prevalent HPV types, including 18 HR-HPV genotypes of HPV-16, −18, −26, −31, −33, −35, −39, −45, −51, −52, −53, −56, −58, −59, −66, −68, −73 and −82, and 3 LR-HPV genotypes of HPV-6, −11 and −81 (equivalent to CP8304). A total of eight reactions per sample were performed simultaneously. Among them, the reactions A, B, C, D, E, F and G were prepared to simultaneously detect and differentiate HPV-16/-18/-31, HPV-59/-66/-53, HPV-33/-58/-45, HPV-56/-52/-35, HPV-68/-51/-39, HPV-73/-26/-82 and HPV-6/-11/-81, respectively. Meanwhile, human TOP3, a single-copy gene encoding DNA topoisomerase III, was amplified in the reaction H as a control for determining relative number of viral copies in a given sample [19].

PCR amplification was conducted in a total reaction volume of 20 μL, which comprised 2 μL DNA samples (up to 50 ng), 10 μL Platinum Quantitative PCR SuperMix-UDG (Invitrogen), 10 pmol of each primer, and 1–5 pmol of each probe (FAM™, VIC^®^ and ROX™ dye). To prevent reamplification of carry-over PCR products, all reactions with Uracil-DNA-Glycosylase (UDG) were pre-incubated at 50 °C for 5 min, followed by an initial denaturation at 95 °C for 10 min, which also inactivates UDG but activates the DNA polymerase, and 45 cycles at 95 °C for 10 s, 58 °C for 40 s. PCR was performed on an ABI Prism 7500 Detection System (Applied Biosystems).

Perfectus Software v1.0, which was used for genotyping and quantitative analysis of HPV nucleic acid (Bioperfectus Limited Corp., China), was applied for quantitative analyses of HPV-16 and -18 viral loads.

### Sequencing

Products of HPV L1 gene amplified from samples by nested PCR using type-specific primers were purified with a QIAquick PCR Purification Kit (Qiagen, Hilden, Germany) as described by the manufacturer’s instructions, and sequenced by Sangon Biotech Co., Ltd (Shanghai, China). Resulting DNA sequences were compared with the sequences of known HPV types using the basic local alignment search tool from the NCBI website (http://www.ncbi.nlm.nih.gov/BLAST).

### Statistical analysis

Cohen’s kappa value (k) was calculated to assess the degree of agreement between results achieved by BMRT HPV PCR assay and HPV GenoArray test. Kappa values of 0–0.2, 0.21–0.4, 0.41–0.6, 0.61–0.8, 0.81–0.99, and 1.0 indicate poor, slight, moderate, substantial, almost perfect and perfect agreement, respectively. *P* values were calculated by Friedman Test. *P* values <0.05 were considered to be statistically significant.

The accuracy measures of the BMRT HPV PCR assay for detecting 21 HPVs, including sensitivity, specificity, positive predictive value (PPV), negative predictive value (NPV) and their relative 95 % confidence intervals (95 % CI) were determined according to sequencing results of PCR products.

Simultaneously, the accuracy measures for predicting CINs in HPV-16 infected patients by viral loads were stratified according to cytological and colposcopy grade. Four patient groups were set up, in which group 1 represents negative for intraepithelial lesion or malignancy (NILM), normal cytology and cervicitis; group 2 includes low-grade cervical intraepithelial neoplasia histology and observation of atypical squamous cells of undetermined significance (ASCUS) or low-grade squamous intraepithelial lesions (LSIL); group 3 is assigned for high-grade intraepithelial neoplasia or worse (CINII+) and high grade squamous intraepithelial lesions (HSIL); group 4 is for cancer. In order to calculate the prediction accuracy measures of BMRT HPV PCR assay for the cytology and colposcopy diagnoses, only HPV-16 positive cases in the four different thresholds were included. *P* values were calculated by the Kruskal-Wallis test. For statistical analysis, the cytological and histological diagnosis was split between negative (CINI-III) and positive (cancer). Receiver operating characteristic (ROC) curve was constructed to find the clinical cutoff value, relative sensitivity and specificity of the BMRT HPV PCR assay. All statistical calculations were performed using the SPSS version 18.0 (SPSS Inc, Chicago, IL, USA).

## Results

### Concordance rate of the BMRT HPV PCR assay with the HPV GenoArray test

Results of infection status in both the multiple and single HPV positive samples were organized in a 2-by-2 cross-tabulation for each HPV type, by classifying detection results of each sample as positive or negative for both of the BMRT HPV PCR assay and the HPV GenoArray test (Table 1). The overall HPV positive and negative coincidence rates between the two tests were 89.8 % and 97.0 %, respectively; the total concordant coincidence was 96.6 % yielding a kappa value of 0.760. For detections of individual HPV types, the positive coincidence rate of the two methods was 100 % for HPV-59, HPV-68 and HPV-51. An almost perfect agreement were obtained between the two methods for detection of HPV-16 (*k* = 0.844), HPV-18 (*k* = 0.881) and HPV-58 (*k* = 0.809), respectively. And a slight agreement was obtained for HPV-56, which showed the lowest kappa value of 0.284. The discordant results were mainly caused by more HPV-positive samples detected by the BMRT HPV PCR assay than those detected by the HPV GenoArray test.

**Table 1:** Concordance rate of the BMRT HPV PCR assay with the HPV GenoArray test

Type^a^	BMRT+/ GenoArray+	BMRT+/ GenoArray-	BMRT-/ GenoArray+	BMRT-/ GenoArray-	Positive coincidence rate	Negative coincidence rate	Total coincidence rate	Kappa value
HPV16	334	32	31	420	91.5 %	92.9 %	92.3 %	0.844
HPV18	129	10	18	660	87.8 %	98.5 %	96.6 %	0.881
HPV31	23	13	2	779	92.0 %	98.4 %	98.2 %	0.745
HPV59	16	19	0	782	100.0 %	97.6 %	97.7 %	0.617
HPV66	23	10	2	782	92.0 %	98.7 %	98.5 %	0.786
HPV53	37	20	2	758	94.9 %	97.4 %	97.3 %	0.757
HPV33	30	10	7	770	81.1 %	98.7 %	97.9 %	0.768
HPV58	73	13	17	714	81.1 %	98.2 %	96.3 %	0.809
HPV45	13	7	1	796	92.9 %	99.1 %	99.0 %	0.760
HPV56	12	53	2	750	85.7 %	93.4 %	93.3 %	0.284
HPV52	44	65	6	702	88.0 %	91.5 %	91.3 %	0.513
HPV35	9	15	1	792	90.0 %	98.1 %	98.0 %	0.521
HPV68	18	31	0	768	100.0 %	96.1 %	96.2 %	0.522
HPV51	9	33	0	775	100.0 %	95.9 %	96.0 %	0.341
HPV39	14	17	1	785	93.3 %	97.9 %	97.8 %	0.599
HPV6	51	22	3	741	94.4 %	97.1 %	96.9 %	0.787
HPV11	21	13	3	780	87.5 %	98.4 %	98.0 %	0.714
HPV81	29	22	4	762	87.9 %	97.2 %	96.8 %	0.675
Total	885	405	100	13316	89.8 %	97.0 %	96.6 %	0.760

Note: ^a^Only types detected in both methods were included

### Accuracy of the BMRT HPV PCR assay compared with sequencing results

As shown in Table 2, the sensitivity, specificity and concordance rate (accuracy) of the BMRT HPV PCR assay was 98.4 %, 99.6 % and 99.6 % by comparing with sequencing results, respectively. For detection of individual HPV types, 100 % sensitivity was achieved by the method for detections of 13 HPV genotypes, including HPV-59, −66, −53, −45, −56, −35, −68, −51, −39, −82, −26, −73 and −11, and 100 % specificity and accuracy for HPV-66, −45, −82 and −73. The accuracies for detections of HPV-18, −58 and −52 by BMRT HPV PCR assay were 99.4 %, 99.4 % and 99.1 %, respectively. However, the accuracy for detection of HPV-16, the most common HPV type, was the lowest one (98.8 %), even though it had the highest number of samples compared to the other HPV types. Compared with the sequencing results, the overall PPV and NPV of the BMRT HPV PCR assay was 95.7 % and 99.9 %, respectively. Identical with the specificity and sensitivity, the PPV of the BMRT HPV PCR assay for detections of HPV-66, −45, −82 and −73 were 100 %, and the NPV for detections of HPV-59, −66, −53, −45, −56, −35, 68, 51, 39, 82, 26, 73 and 11 were 100 %.

**Table 2:** Accuracy of the BMRT HPV PCR assay compared with sequencing from all the 817 samples

Type	+^a^/+^b^	+/-	-/+	-/-	Sensitivity	Specificity	False positive rate	False negative rate	Concordance rate	PPV	NPV	*k*	95 % CI
HPV16	361	7	3	446	99.2 %	98.5 %	1.5 %	0.8 %	98.8 %	98.1 %	99.3 %	0.975	0.960–0.991
HPV18	138	1	4	674	97.2 %	99.9 %	0.1 %	2.8 %	99.4 %	99.3 %	99.4 %	0.979	0.960–0.997
HPV31	35	1	1	780	97.2 %	99.9 %	0.1 %	2.8 %	99.8 %	97.2 %	99.9 %	0.971	0.931–1.011
HPV59	32	3	0	782	100.0 %	99.6 %	0.4 %	0.0 %	99.6 %	91.4 %	100.0 %	0.953	0.901–1.006
HPV66	33	0	0	784	100.0 %	100.0 %	0.0 %	0.0 %	100.0 %	100.0 %	100.0 %	1	~
HPV53	54	3	0	760	100.0 %	99.6 %	0.4 %	0.0 %	99.6 %	94.7 %	100.0 %	0.971	0.938–1.004
HPV33	39	1	3	774	92.9 %	99.9 %	0.1 %	7.1 %	99.5 %	97.5 %	99.6 %	0.949	0.896–0.999
HPV58	85	1	4	727	95.5 %	99.9 %	0.1 %	4.5 %	99.4 %	98.8 %	99.5 %	0.968	0.940–0.996
HPV45	20	0	0	797	100.0 %	100.0 %	0.0 %	0.0 %	100.0 %	100.0 %	100.0 %	1	~
HPV56	56	9	0	752	100.0 %	98.8 %	1.2 %	0.0 %	98.9 %	86.2 %	100.0 %	0.920	0.868–0.972
HPV52	104	5	2	706	98.1 %	99.3 %	0.7 %	1.9 %	99.1 %	95.4 %	99.7 %	0.963	0.935–0.990
HPV35	21	3	0	793	100.0 %	99.6 %	0.4 %	0.0 %	99.6 %	87.5 %	100.0 %	0.932	0.854–1.008
HPV68	48	1	0	768	100.0 %	99.9 %	0.1 %	0.0 %	99.9 %	98.0 %	100.0 %	0.989	0.968–1.011
HPV51	40	2	0	775	100.0 %	99.7 %	0.3 %	0.0 %	99.8 %	95.2 %	100.0 %	0.974	0.939–1.010
HPV39	30	1	0	786	100.0 %	99.9 %	0.1 %	0.0 %	99.9 %	96.8 %	100.0 %	0.983	0.950–1.016
HPV82	12	0	0	805	100.0 %	100.0 %	0.0 %	0.0 %	100.0 %	100.0 %	100.0 %	1	~
HPV26	2	1	0	814	100.0 %	99.9 %	0.1 %	0.0 %	99.9 %	66.7 %	100.0 %	0.799	0.415–1.184
HPV73	4	0	0	813	100.0 %	100.0 %	0.0 %	0.0 %	100.0 %	100.0 %	100.0 %	1	~
HPV6	70	3	1	743	98.6 %	99.6 %	0.4 %	1.4 %	99.5 %	95.9 %	99.9 %	0.970	0.940–0.999
HPV11	26	8	0	783	100.0 %	99.0 %	1.0 %	0.0 %	99.0 %	76.5 %	100.0 %	0.862	0.767–0.956
HPV81	45	6	2	764	95.7 %	99.2 %	0.8 %	4.3 %	99.0 %	88.2 %	99.7 %	0.913	0.854–0.973
Total	1255	56	20	15826	98.4 %	99.6 %	0.4 %	1.6 %	99.6 %	95.7 %	99.9 %	0.968	0.961–0.975

Note: ^a^indicates the results of the BMRT HPV PCR assay, and ^b^indicates the results of the sequencing

Compared with sequencing results, the HPV genotyping by the BMRT HPV PCR assay showed perfect agreement (*k* = 0.968; 95%CI, 0.961–0.975); Meanwhile, the accuracy of the BMRT HPV PCR assay was 91.6 % (95 % CI, 88.8–94.0, n = 467) and 90.8 % (95 % CI, 87.3–93.7, n = 350) for samples with single and multiple infections, respectively. For detections of individual HPV types, the BMRT HPV PCR assay and sequencing showed almost perfect agreement.

### Analyses of discordant HPV typing results between the BMRT HPV PCR assay and sequencing by comparing to the historical diagnoses and infection states of the cases

The discordant HPV typing results between the BMRT HPV PCR assay and sequencing were estimated for single and multiple infections (Table 3). For detections of all HPV types, total 76 cases showed discordant in BMRT HPV PCR assay and sequencing results. Among the 76 discordant cases, 20 cases (6 cases of single infection and 14 of multiple infections) were detected by the sequencing but not by the BMRT HPV PCR assay. In contrast, the other 56 cases (32 cases of single infection and 24 of multiple infections) were identified by the BMRT HPV PCR assay but not by the sequencing.

**Table 3:** Analyses of discordant HPV typing results between the BMRT PCR assay and sequencing by comparing to the historical diagnoses and infection states of the cases

Type	Numbers of BMRT negative/sequencing positive			Numbers of BMRT positive/sequencing negative		
	Infection states		Historical diagnoses	Infection states		Historical diagnoses
	Total	(Single + multiple^a^)	(N + CINI+ CINII-III + cancer)	Total	(Single + multiple^a^)	(N + CINI+ CINII-III + cancer)
HPV16	3	(2 + 1)	(3 + 0 + 0 + 0)	7	(0 + 7)	(7 + 0 + 0 + 0)
HPV18	4	(0 + 4)	(1 + 2 + 1 + 0)	1	(0 + 1)	(1 + 0 + 0 + 0)
HPV31	1	(1 + 0)	(0 + 0 + 1 + 0)	1	(0 + 1)	(1 + 0 + 0 + 0)
HPV59	0			3	(1 + 2)	(2 + 0 + 1 + 0)
HPV53	0			3	(0 + 3)	(0 + 3 + 0 + 0)
HPV33	3	(1 + 2)	(3 + 0 + 0 + 0)	1	(0 + 1)	(1 + 0 + 0 + 0)
HPV58	4	(0 + 4)	(4 + 0 + 0 + 0)	1	(0 + 1)	(1 + 0 + 0 + 0)
HPV56	0			9	(1 + 8)	(7 + 0 + 1 + 1)
HPV52	2	(1 + 1)	(2 + 0 + 0 + 0)	5	(1 + 4)	(3 + 0 + 1 + 1)
HPV35	0			3	(0 + 3)	(0 + 0 + 2 + 1)
HPV68	0			1	(1 + 0)	(1 + 0 + 0 + 0)
HPV51	0			2	(0 + 2)	(1 + 0 + 1 + 0)
HPV39	0			1	(0 + 1)	(1 + 0 + 0 + 0)
HPV26	0			1	(0 + 1)	(1 + 0 + 0 + 0)
HPV6	1	(0 + 1)	(1 + 0 + 0 + 0)	3	(0 + 3)	(1 + 1 + 1 + 0)
HPV11	0			8	(1 + 7)	(3 + 1 + 4 + 0)
HPV81	2	(1 + 1)	(2 + 0 + 0 + 0)	6	(0 + 6)	(6 + 0 + 0 + 0)
Total	20	(6 + 14)	(16 + 2 + 2 + 0)	56	(5 + 51)	(37 + 5 + 11 + 3)

Note: N means normal in historical diagnosis. ^a^show the numbers of multiple infection cases, in which additional HPV types were detected by the indicated (Positive) method

Among the 65 multiple infected cases, which determined by either the BMRT HPV PCR assay or sequencing, 51 cases were positive for at least one additional HPV type by the BMRT HPV PCR assay than sequencing. While, among the 23 discordant cases of patients with CINs and cancer, 19 cases were detected by the BMRT HPV PCR assay, but only 4 cases with CINs were detected by sequencing. These results indicate that the BMRT HPV PCR assay may be more suitable for detecting multiple infections in women with pathological lesions than sequencing method.

### Relationship between relative DNA loads of HPV-16 or HPV-18 and histomorphological findings of infected women

**Fig. 1:**
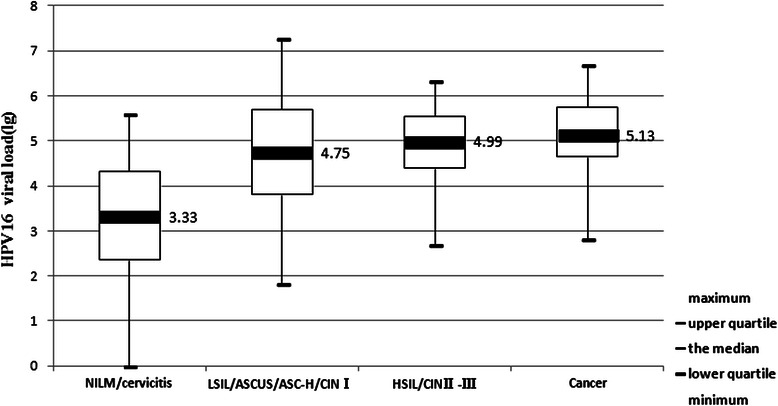
The relationship between HPV - 16 viral load and the histopathology of cervical samples

**Fig. 2:**
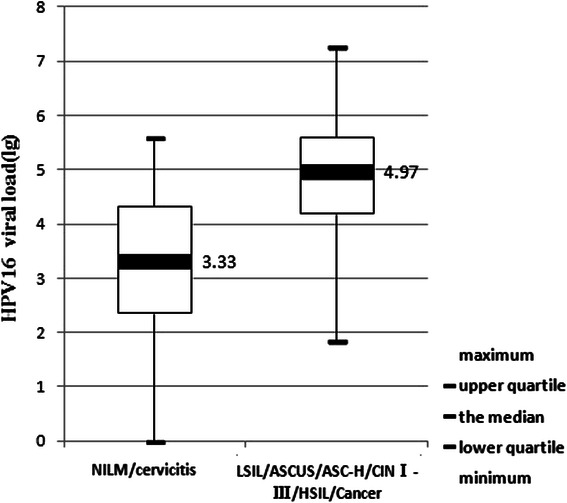
Comparison of HPV - 16 viral loads between NILM/cervicitis and ASCUS/CIN I-III/cancer

All cervical scrapes from HPV-16 and HPV-18 positive women were taken in the course of a colposcopic examination and biopsies. Altogether, 313 HPV-16 and 130 HPV-18 positive patients fulfilled this criterion. Among the 313 HPV-16 positive patients, 171 were NILM/cervicitis, 56 were LSIL/ASCUS/ASC-H/CINI, 63 were HSIL/CINII-III and 23 were cervical cancer. The relationships between relative loads of HPV-16 DNA (copies per 10,000 cells) in corresponding cervical scrapes and the histopathological findings of the patients were analyzed. As shown in Fig. 1, the median viral load (lg) in patients with NILM/cervicitis, LSIL/ASCUS/ASC-H/CINI, HSIL/CINII-III and cancer were 3.33, 4.75, 4.99 and 5.13, respectively. Moreover, pairwise comparisons among the four groups by Kruskal-Wallis test showed no significant differences of viral loads in samples between the two CIN groups, as well as between each of the two CIN groups and the cancer group. However, highly marked differences were observed when comparing the group presenting NILM/cervicitis to either all grades of CIN (*p* < 0.001), or to cervical carcinomas group (*p* < 0.001) by Mann–Whitney *U* test. As shown in Fig. 2, the median viral loads (copies per 10,000 cells) in patients with NILM/cervicitis and ASCUS/CINI-III/cancer were 3.33 and 4.97, respectively.

To discriminate the group of NILM and cervicitis from CINI-III and cancer, the area under the curve (AUC) for HPV-16 viral load was calculated for two endpoints of CINI and greater, and of CINII and greater. For HPV-16, the AUCs were 0.827 for CINI and greater, and 0.786 for CINII and greater. The optimal cutoff value of 16,600 copies per 10,000 cells was selected. This value corresponds to 74.6 % sensitivity and 72.5 % specificity for predicting CINI and greater, to 80.2 % sensitivity and 63.0 % specificity for predicting CINII and greater.

**Fig. 3:**
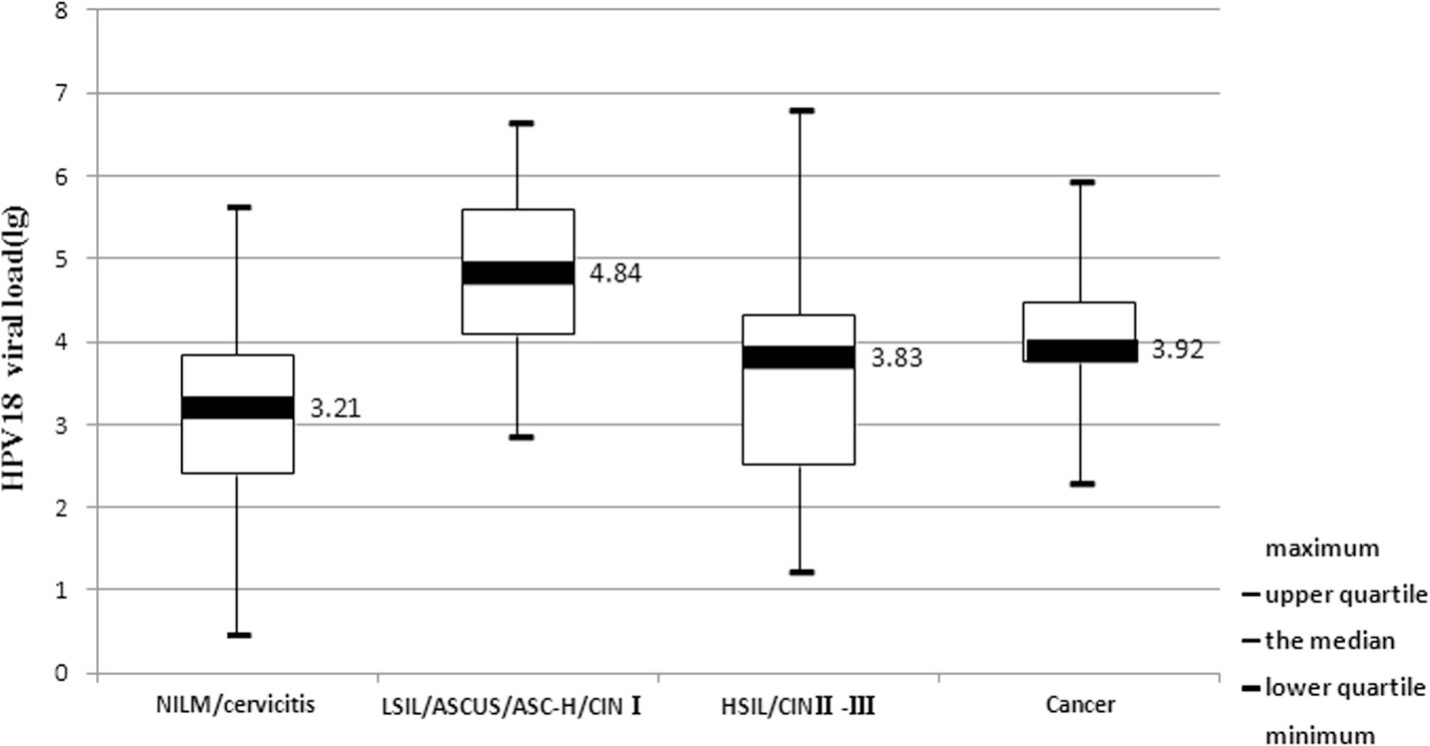
The relationship between HPV - 18 viral load and the histopathology of cervical samples

**Fig. 4:**
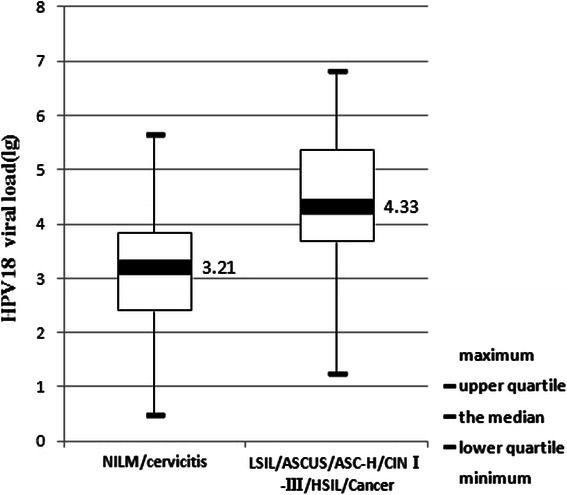
Comparsion of HPV - 18 viral loads between NILM/cervicitis and ASCUS/CIN I-III/cancer

Among 130 HPV-18 positive patients, 81 were negative in histological diagnosis (including normal, infusorian and cervicitis), 27 were LSIL/ASCUS/ASC-H/CINI, 14 were HSIL/CINII-III, and 8 were cervical cancer. The relative levels of HPV-18 DNA loads (copies per 10,000 cells) in corresponding cervical scrapes were calculated to correlate them with the histopathological findings of infected patients (Fig. 3). The median viral load (lg) in patients with diagnosis of NILM/cervicitis, LSIL/ASCUS/ASC-H/CINI, HSIL/CINII-III and cancer were 3.21, 4.84, 3.83 and 3.92, respectively. Moreover, pairwise comparisons among the four groups by Kruskal-Wallis test showed highly significant difference of viral loads in samples between the group presenting NILM/cervicitis and the group of LSIL/ASCUS/ASC-H/CINI (*p* < 0.001). Unlike those of HPV16, the viral loads of HPV-18 were less associated with progress of CINII-III. Similar to those of HPV-16, the significant differences of HPV-18 viral loads were observed when comparing the group presenting NILM/cervicitis to either all grades of CIN (*p* < 0.001), or to cervical carcinomas group (*p* < 0.001) by Mann–Whitney *U* test. As shown in Fig. 4, the median viral loads (copies per 10,000 cells) in patients with NILM/cervicitis and ASCUS/CINI-III/cancer were 3.21 and 4.31, respectively.

## Discussion

In clinical screening of HPV-infected women, accurate HPV genotyping has become an important prognostic indicator for monitoring persistent HPV infection, which is the strong causality of high grade cervical intraepithelial neoplasia [18, 20]. In our hospital, HPV DNA is routinely detected in Cervical Cancer Screening Program by the HPV GenoArray method, which allows for genotyping of 13 HR types, 5 LR types and 3 other types commonly found in China [18]. However, the HPV GenoArray method does not provide quantitative information on detected HPV DNA. Evidence is accumulating that HPV quantification may be useful as genotyping for patient management in the future. In order to solve this problem, a multiplex real time assay was designed for simultaneous genotyping and quantification of the 18 most frequent cancer related HR-HPV types and 3 LR-HPV types of HPV-6, −11 and −81. A recent cross-sectional study showed that these types mentioned above were the predominant HPV types in women with high-grade lesions [21].

Quantitative real-time PCR methods are considered to be the gold standard for HPV load assessment, but these have not been developed and validated for the genotyping and quantifying wide spectrum of carcinogenic HPV types often encountered in cervical samples [22] . HPV DNA viral load, usually estimated as the amount of HPV genome copies per cell, has been variably associated with cervical disease, and appears to have an overall specificity to differentiate normal cytology from abnormal cytology [23]. A novel application of the BMRT HPV PCR assay for the HPV genotyping and quantifying is described. The BMRT HPV PCR assay is a multiplex gene analysis platform, offers a high sensitive, cost-effectiveness and high throughput assay that allows the rapid and specific detection of 21 high-risk and low-risk HPV genotypes in conjunction with BMRT genetic analysis system. Each pair of HPV-specific primers only generated a single peak for each HPV genotype in addition to the internal control peak. Analyses of 817 specimens using the BMRT PCR assay and the HPV GenoArray test demonstrated that the BMRT PCR assay had comparable sensitivity and specificity to HPV GenoArray test (Table 2). In this study, a 96.6 % of total coincidence rate and a substantial almost perfect agreement (k = 0.760) for genotyping 18 HPV types, which are capable detected in both the methods, was achieved between the BMRT HPV PCR assay and the HPV GenoArray test. The BMRT HPV PCR assay was proved to be highly specific for typing the 21 HPV types. This is a particular note since both assays differ considerably in their design. The HPV GenoArray test is based on amplification by a pair of consensus primers in a highly conserved region of the L1 gene and reverse hybridization to the type-specific probes, whereas the BMRT HPV PCR assay utilizes type-specific primers and TaqMan probes to amplify the sequences within the L1 region. Numerous studies have been showed that the GenoArray test is a highly reproducible assay with an excellent clinical sensitivity for HPV types and is thus considered to be adequate for comparison [18, 20]. Most of the discordant results detected by the two methods were seen in cases with multiple infections. Generally, agreement is relatively poor between various assays for genotyping multiple HPV infections. A recent study, which evaluated different multiplex HPV PCR assays for identification of low prevalent HPV types, revealed only moderate inter-assay agreement in cases of single HPV infections and poor agreement in cases with multiple HPV infections [24].

DNA sequencing is the “gold standard” method for accurate HPV genotyping, in which HPV DNA is amplified with specific primers followed by sequencing. It is desirable to evaluate the accuracy of any HPV genotyping method on the basis of the sequencing results [25, 26]. In this study, a perfect agreement (*k* = 0.968; 95 % CI, 0.961–0.975) for HPV genotyping between the BMRT HPV PCR assay and sequencing was obtained. Compared with sequencing method, the sensitivity, specificity and accuracy of the BMRT HPV PCR assay was 98.4 %, 99.6 % and 99.6 %, respectively. This result indicates that HPV genotype could be successfully identified by the BMRT HPV PCR assay with high accuracy.

The BMRT HPV PCR assay was demonstrated to have good sensitivity, specificity, accuracy, PPV and NPV compared to the sequencing method. Among detections of the 21 HPV genotypes, detections of 13 HPV genotypes by the BMRT HPV PCR assay showed 100 % sensitivity and NPV value, detections of 4 HPV genotypes showed 100 % specificity and PPV value. All HPV infections in patients with CINs and cancer were successfully detected by the BMRT HPV PCR assay except for 2 cases of patients with CINII-III and 2 with CIN. Of the 817 HPV positive samples (Detections of the 21 HPV genotypes were performed in each sample), only 20 cases were positive (in 6 single infection cases and for additional types in 14 multiple infection cases) in the sequencing test but negative in the BMRT HPV PCR assay. However, among these 20 cases only two were diagnosed as CINII-III and the others were diagnosed as normal, cervicitis and CINI. This result suggests that the BMRT PCR method could successfully detect HPV infections and identify HPV genotypes with a high degree of accuracy.

In this study, the results showed that the median relative levels of HPV-16 DNA do not vary by more than 2-folds irrespective of the severity of CIN. There is also no statistical significance of viral loads in samples between the two CIN groups, as well as between each of the two CIN groups and the cancer group by Kruskal-Wallis test. The data indicate that viral load does not correlate with disease progression within the CIN spectrum. However, highly significant differences of viral loads were achieved when comparing the group lacking CIN with the group comprising of all CIN (*p* < 0.001) or with the cancer group (*p* < 0.001). A significant decrease of viral load in liquid based cytology samples from cervical carcinoma patients compared to those from CIN patients was reported by Yoshida et al [27]. However, our quantitative data showed that the relative number of HPV genomic copies within a histological entity can vary within the upper and lower quartile of the box plot (Fig. 1). The differences are several log-folds when considering the extreme values of each group. Similar observations were reported in a recent study in which the variation in HPV-16 viral load within different histological grades of cervical neoplasia were evaluated [28]. It is therefore likely that changes in viral load over time, rather than a single measurement, might be predictive for disease progression or clearance [17].

In contrast to HPV-16, viral loads of HPV-18 were less associated with CINII-III or cancer. This observation may be due to the different viral activity between HPV-18 and HPV-16, or a higher proportion of HPV-18 infections in patients with glandular lesions that are more difficult to sample and hence are prone to false-negative results. Our data suggest that careful attention should be paid to HPV-18 as the same as to HPV-16, but for a different reason. Specifically, the possible use of HPV-18 typing to improve the detection of cytologically occult lesions should be formally evaluated. Extended analyses, accounting for multiple carcinogenic infections, are necessary to evaluate the role of viral load for other carcinogenic HPV types.

From an economic point, the BMRT HPV PCR assay costs less and is faster (less than 2.5 h, including DNA extraction). Moreover, the real-time PCR assay enables reliable quantifications of the target DNA. Our approach provides a potential for viral load assessment for 21 types in parallel (not only HPV-16 and HPV-18). Additionally, the assay will be useful to evaluate the clinical relevance of viral persistence at the genotype level, monitor disease recurrence, and examine the effects of widespread vaccination on prevalent HPV types in the future.

## Conclusion

The BMRT assay showed a similar clinical performance for genotyping compared with the HPV GenoArray test and seemed to be a good alternative approach for HR-HPV testing,due to its high level of automation and ability to quantify HPV - 16, HPV - 18 and other HR-HPV. Moreover, the BMRT assay could potentially promote patient management by risk stratification of cytological abnormal populations. This new assay could be a useful tool for both primary screening of cervical cancer and the triage of women with abnormal cytology.

## Abbreviations

HPV: Human papillomavirus
NILM: Intraepithelial lesion or malignancy
BMRT: BioPerfectus Multiplex Real Time
CIN: Cervical intraepithelial neoplasia grade
RLB: Reverse line blot
LR: Low risk
GAPDH: Glyceraldehyde 3-phosphate dehydrogenase
UDG: Uracil-DNA-Glycosylase
ASCUS: Atypical squamous cells of undetermined significance
LSIL: Low-grade squamous intraepithelial lesions
HSIL: High grade squamous intraepithelial lesions
AUC: Area under the curve
